# Age-Dependent Impairment of Eyeblink Conditioning in Prion Protein-Deficient Mice

**DOI:** 10.1371/journal.pone.0060627

**Published:** 2013-04-10

**Authors:** Yasushi Kishimoto, Moritoshi Hirono, Ryuichiro Atarashi, Suehiro Sakaguchi, Tohru Yoshioka, Shigeru Katamine, Yutaka Kirino

**Affiliations:** 1 Laboratory of Neurobiophysics, Graduate School of Pharmaceutical Sciences, The University of Tokyo, Tokyo, Japan; 2 Laboratory for Motor Learning Control, RIKEN Brain Science Institute, Wako, Japan; 3 Department of Molecular Microbiology and Immunology, Nagasaki University Graduate School of Biomedical Sciences, Nagasaki, Japan; 4 Division of Molecular Neurobiology, The Institute for Enzyme Research, The University of Tokushima, Tokushima, Japan; 5 Center of Excellence for Environmental Medicine, Kaohsiung Medical University, Kaohsiung, Taiwan; 6 Center for International Collaborative Research, Nagasaki University, Nagasaki, Japan; Dulbecco Telethon Institute and Mario Negri Institute for Pharmacological Research, Italy

## Abstract

Mice lacking the prion protein (PrP^C^) gene (*Prnp*), Ngsk *Prnp* ^0/0^ mice, show late-onset cerebellar Purkinje cell (PC) degeneration because of ectopic overexpression of PrP^C^-like protein (PrPLP/Dpl). Because PrP^C^ is highly expressed in cerebellar neurons (including PCs and granule cells), it may be involved in cerebellar synaptic function and cerebellar cognitive function. However, no studies have been conducted to investigate the possible involvement of PrP^C^ and/or PrPLP/Dpl in cerebellum-dependent discrete motor learning. Therefore, the present cross-sectional study was designed to examine cerebellum-dependent delay eyeblink conditioning in Ngsk *Prnp* ^0/0^ mice in adulthood (16, 40, and 60 weeks of age). The aims of the present study were two-fold: (1) to examine the role of PrP^C^ and/or PrPLP/Dpl in cerebellum-dependent motor learning and (2) to confirm the age-related deterioration of eyeblink conditioning in Ngsk *Prnp* ^0/0^ mice as an animal model of progressive cerebellar degeneration. Ngsk *Prnp* ^0/0^ mice aged 16 weeks exhibited intact acquisition of conditioned eyeblink responses (CRs), although the CR timing was altered. The same result was observed in another line of PrP^c^-deficient mice, ZrchI *PrnP* ^0/0^ mice. However, at 40 weeks of age, CR incidence impairment was observed in Ngsk *Prnp* ^0/0^ mice. Furthermore, Ngsk *Prnp* ^0/0^ mice aged 60 weeks showed more significantly impaired CR acquisition than Ngsk *Prnp* ^0/0^ mice aged 40 weeks, indicating the temporal correlation between cerebellar PC degeneration and motor learning deficits. Our findings indicate the importance of the cerebellar cortex in delay eyeblink conditioning and suggest an important physiological role of prion protein in cerebellar motor learning.

## Introduction

Sheep scrapie, bovine spongiform encephalopathy (BSE), and Creutzfeldt–Jacob disease (CJD) in humans are variants of prion diseases, which are caused by infectious agents named prions [1]–[5]. Prion diseases may be expressed as genetic, infectious, or sporadic disorders, all of which involve modification of the normal cellular form of the prion protein (PrP^C^). The predominant symptoms are progressive dementia and motor dysfunction such as ataxia [6]–[7]. To investigate the physiological role of PrP^C^, several independent PrP^C^-deficient mouse lines have been developed [8]–[12].

PrP^C^-deficient mice generated by some of us (Ngsk *Prnp* ^0/0^ mice) in which the functional PrP gene (*Prnp*), including the entire PrP-coding sequence of exon 3 and a part of intron 2 (900 bp), was deleted showed progressive symptoms of ataxic gait and hindquarter tremors late in life [13]. Late-onset ataxia is also observed in 2 other independently derived lines of *Prnp* ^0/0^ mice [9], [11]. In Ngsk *Prnp* ^0/0^ mice, Purkinje cell (PC) death began at approximately 30–40 weeks of age [13]–[15]. The other 2 lines of PrP^C^-deficient mice, including ZrchI *Prnp* ^0/0^ mice, do not exhibit cerebellar degeneration [8], [10]. Ectopic expression of the novel locus *Prnd*, which is 16 kb downstream of *Prnp* and encodes a 179-residue PrP-like protein Doppel (PrPLP/Dpl), has been observed in the brain of Ngsk *Prnp* ^0/0^ mice, but not in ZrchI *Prnp* ^0/0^ brains, and has been implicated in the cerebellar degeneration of Ngsk *Prnp* ^0/0^ mice [11], [16], [17]. However, PrP^C^ may be partially involved in long-term survival of PCs because Ngsk *Prnp* ^0/0^ mice were rescued from PC degeneration by introduction of a transgene encoding wild-type mouse PrP^C^ [15]. Cerebellar degeneration thus appears to require both functional loss of PrP^C^ and cerebellar overexpression of PrPLP/Dpl [18].

PrPLP/Dpl is a putative membrane glycoprotein sharing 23% identity with the PrP^C^ primary amino acid sequence structure [11], [19], [20]. The function and expression pattern of PrPLP/Dpl have been investigated in the past decade [17], [21]–[26]. However, the molecular mechanism underlying the neuronal degeneration induced by ectopic expression of PrPLP/Dpl is unclear, although some hypotheses have been proposed [27]–[29].

Eyeblink conditioning is one of the best-characterized behavioral models of associative learning in mammals [30]–[32]. In particular, the standard delay paradigm in which the conditioned stimulus (CS) and unconditioned stimulus (US) are continuous has been used for assessing cerebellum-dependent motor learning in a variety of mammalian species. The neural circuit involved in the response is well defined. The memory trace is considered to be localized in the cerebellar cortex and/or cerebellar deep nuclei, ipsilateral to the trained eye [30], [33]–[38], even if the relative importance of the cerebellar cortex versus that of the cerebellar deep nuclei remains controversial [37], [39]–[45].

Because PrP^C^ is highly expressed in cerebellar neurons including PCs, granule cells, and deep nuclei [46]–[48], it may be involved in cerebellar synaptic function and cerebellum-dependent cognitive function. Therefore, investigation of the cerebellar motor learning in protein-deficient mice is important. However, no studies have been conducted to investigate the possible involvement of PrP^C^ and/or PrPLP/Dpl in cerebellum-dependent discrete motor learning such as adaptation of the vestibulo-ocular reflex (VOR) or eyeblink conditioning. Thus, the present study was designed to test delay eyeblink conditioning, in which the cerebellum is believed to play important roles for both acquisition and timing of the conditioned response (CR) [31], [32], [40], [49]–[53], in Ngsk *Prnp* ^0/0^ mice in adulthood (16, 40, and 60 weeks of age). The aims of the present study were two-fold: (1) to examine the role of PrP^C^ and/or PrPLP/Dpl in cerebellum-dependent motor learning and (2) to confirm the age-related deterioration of eyeblink conditioning in Ngsk *Prnp* ^0/0^ mice as an animal model of progressive cerebellar degeneration.

## Materials and Methods

### Ethics Statement

All animal protocols were approved by the Animal Experiment Ethics Committee at the University of Tokyo, and the mice were cared for in accordance with the University of Tokyo Guidelines for the Care and Use of Laboratory Animals. We used the minimum number of animals for these experiments, and care was taken to minimize pain.

### Subjects

Ngsk *Prnp* ^0/0^ mice were generated as described previously [12], [13]. Male F3 Ngsk *Prnp* ^0/0^ mice were crossed with female C57BL/6J mice (purchased from CLEA Japan, Tokyo, Japan), producing F4 heterozygous mice (*Prnp* ^+/0^ mice). Mutant mice (Ngsk *Prnp* ^0/0^) and their littermate controls (Ngsk *Prnp* ^+/+^) were derived by intercrossing F4 Ngsk *Prnp* ^+/0^ males and females. Genotypes were confirmed by polymerase chain reaction (PCR) amplification of tail-extracted genomic DNA from each mouse. The specific primers for the mouse *Prnp* gene are 5′-CCGCTACCCTAACCAAGTGT-3′ and 5′-CCTAGACCACGAGAATGCGA-3′ (generates a 346-bp PCR fragment). The neomycin-resistant gene primers are 5′-GGTGCCCTGAATGAACTGCA-3′ and 5′-GGTAGCCGGATCAAGCGTAT-3′, resulting in a 227-bp PCR fragment.

We obtained 46 Ngsk *Prnp* ^+/+^ (32.6%), 61 Ngsk *Prnp* ^+/0^ (43.3%), and 34 Ngsk *Prnp* ^0/0^ (24.1%) mice. Ngsk *Prnp* ^+/+^ and Ngsk *Prnp* ^0/0^ mice were used for behavioral tests. They were maintained as described until the age of use (16, 40, or 60 weeks). ZrchI *Prnp* ^0/0^ mice were produced as described previously [8] and maintained in 3 genetic backgrounds (C57BL/6J, 129/SvJ, and FVB/Nj) [15] until the age of 18–22 weeks. The 3 wild-type control strains (18–22 weeks of age) were purchased from CLEA Japan (Tokyo, Japan). Before all behavioral experiments, mice were handled extensively by the experimenter to acclimate them.

All mice were maintained in a specific pathogen-free room with controlled humidity (55±5% relative humidity) and temperature (24±2°C) in the School of Pharmaceutical Sciences at The University of Tokyo on a 12-h light:dark cycle with food and water available ad libitum. At the termination of the experiments, mice were sacrificed by cervical dislocation.

### Surgery

Mice were anesthetized using ketamine (80 mg/kg, *i.p.*; Sankyo, Tokyo, Japan) and xylazine (20 mg/kg, *i.p.*; Bayer, Tokyo, Japan). Four Teflon-coated stainless-steel wires (100 µm in diameter; A-M Systems, Sequim, WA, USA) were subcutaneously implanted under the left eyelid. Two wires were used to record the electromyographic (EMG) activity of the orbicularis oculi muscle, which is associated with eyelid closure, and the remaining 2 wires were used to deliver the periorbital shock US.

### Eyeblink Conditioning Procedures

The conditioning apparatus and procedures were similar to previously described methods [54], [55]. At least 3 days were allotted for recovery and 2 days for acclimatization to the conditioning chamber after the surgery. A 352-ms tone (1 kHz, 80 dB) was used as the CS and a 100-ms electrical shock (100-Hz square pulses) was the US. Daily training consisted of 100 trials grouped in 10 blocks. Each block included 1 CS alone (at the tenth trial) and 9 CS–US paired trials. For paired trials, the US was timed to overlap the CS so that the 2 stimuli terminated simultaneously. Intertrial intervals were randomized between 20 s and 40 s, with a mean of 30 s. The US intensity was carefully determined as the minimal voltage required to elicit an eyeblink reflex and was adjusted daily for each animal (under 0.8 mA). Experiments were performed during the light phase of the light:dark cycle in a 10-cm-diameter container placed in a sound- and light-attenuating chamber. All experiments, including surgery, were performed by an experimenter who was blinded to the genotypes of the mice. The spontaneous eyeblink frequency was measured by 100 “no stimulus” trials before the conditioning experiment began, and the startle response to a tone was measured during the first 100 trials of the first delay eyeblink conditioning session.

### Data Analyses

The EMGs were band-pass filtered between 0.15 and 1.0 kHz and fed into a computer with a sampling rate of 10 kHz. Data for each session were processed offline as follows: (i) The maximum amplitude of the EMG signals during a time period of t ±1 ms was calculated and denoted as the EMG amplitude at t. (ii) One-hundred EMG amplitude values for 300 ms were averaged (before CS onset), and the standard deviation (SD) was calculated. (iii) The average value obtained from (ii) +SD was defined as the threshold. (iv) For each trial, EMG amplitude data for 300 ms over the threshold were averaged (before CS onset) and called the Pre value. The Startle value was calculated in the same manner for 30 ms after CS onset. The CR value was calculated from the data for the period 52–252 ms after CS onset in the CS–US paired trials. The time window was extended by 100 ms to obtain the CR value in the CS-only trials. (v) Valid trials were defined as those with Pre and Startle values of less than 10% and 30% of the threshold, respectively. (vi) A trial in which the CR value exceeded 1% of the threshold and was double the Pre value was regarded as a successful CR trial. (vii) The ratio of successful CR trials to valid trials was calculated and denoted as the CR%.

In addition to the CR incidence analyses, the temporal pattern of eyeblink responses were also analyzed by averaged EMG and peak latency histogram analyses. In the EMG amplitude analyses, all 100 trials (including invalid trials) were used on day 3 or 7 [54], whereas only CR trials were used for the peak latency histogram analyses [55]. In the histogram analyses, the relative frequencies of the CR peak were plotted as a function of its latency. The CR on day 7 was binned into time windows of 52.1–102.0, 102.1–152.0, 152.1–202.0, 202.1–252.0, 252.1–302.0, and 302.1–352.0 ms, measured from the CS onset.

### Statistical Analyses

Data were statistically analyzed in Microsoft Excel using a two-tailed Student’s *t*-test (for histogram latency analyses [55]), or repeated-measures ANOVA following a post hoc Scheffé’s test (for CR% analyses [55]–[57]). Differences were considered statistically significant when *p* was less than 0.05.

## Results

### CR Acquisition in 16-week-old Ngsk *Prnp* ^0/0^ Mice

**Figure 1:**
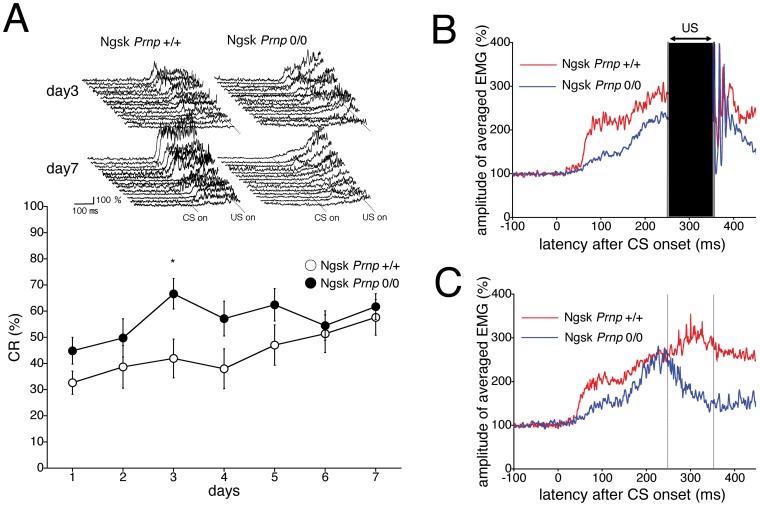
Eyeblink conditioning in 16-week-old Ngsk *Prnp* ^0/0^ mice. (**A**) CR development was calculated using 100 trials/day for each animal during a 7-day session in 16-week-old control (open circle, n = 14) and Ngsk *Prnp* ^0/0^ (experimental mice; closed circle, n = 14) mice. The top inset shows individual response topographies of averaged electromyographs (EMGs) until US onset in the control and experimental mice on days 3 and 7. (**B**) The averaged EMG amplitudes on day 7 of the acquisition session in control (n = 14) and experimental (n = 14) mice. All EMG amplitudes obtained in 1 day (100 trials) were summed, representing the average of the eyelid responses. The CR components of 250–350 ms from CS onset were masked by artifacts caused by the US. (**C**) The averaged EMG amplitudes were evaluated using only 10 CS-only trials on day 7 of the acquisition session. These analyses enable us to indicate EMG patterns in the whole CS period without the US artifacts. Values are mean ± SEM. *p<0.05.

First, delay eyeblink conditioning with the CS and US simultaneously terminated was performed in 16-week-old Ngsk *Prnp* ^0/0^ mice (without apparent histological and motor abnormalities) during 7 consecutive days. Figure 1A shows the development of the averaged percentage of the acquired conditioned response (CR%, an index of learning), which was calculated using 100 trials/day for each animal, in control (*Prnp* ^+/+^) and experimental (Ngsk *Prnp* ^0/0^) mice. The CR% for the 2 genotypic groups progressively increased to approximately 60% during the 7-day acquisition session, although the CR acquisition in experimental mice was moderately higher than in the control mice. A repeated-measures ANOVA revealed no significant group × session interaction effect (*F*_(6,156)_ = 1.299, *p* = 0.26) or group effect (*F*_(1,26)_ = 3.14, *p* = 0.088) during the acquisition session. The top inset in Figure 1A shows individual EMG topographies averaged by 100 trials on days 3 and 7 in both groups. On day 3, experimental mice displayed somewhat higher CR amplitudes than the control mice did. Therefore, we conclude that CR acquisition of eyeblink conditioning was not impaired in experimental 16-week-old mice aged.

No difference was detected between control and experimental mice in the spontaneous eyeblink frequency (13.1±2.1% for control mice and 15.3±2.8% for experimental mice). Furthermore, the relative number of valid trials (81.8%±4.4% for control mice and 79.9%±6.8% for experimental mice) and the frequency of startle responses (5.3%±1.0% for control mice and 6.2%±1.8% for experimental mice) were not significantly different. In addition, no differences were noted between 16-week-old control mice and experimental mice in pseudoconditioning (see Figure S1A and Text S1) or responses to CS (see Figure S2A and Text S1). The experimental mice at the age of 16 weeks exhibited normal performance in rota-rod and fixed-bar tests, indicating that motor coordination was normal in this mutant at this age (data not shown).

### Altered Timing of CR in Ngsk *Prnp* ^0/0^ Mice Aged 16 Weeks

Figure 1B depicts the averaged electromyographic (EMG) amplitudes obtained from control and experimental mice aged 16 weeks on day 7 of the acquisition session. The data suggest that CRs in experimental mice are altered in their timing. In Figure 1B, however, CR components in the range of 250–350 ms from CS onset were masked by US artifacts. Therefore, we re-evaluated the averaged EMG amplitudes by using the 10 CS-only trials on day 7 in the acquisition session (Figure 1C). These analyses enabled us to investigate EMG patterns throughout the CS period without the US artifacts. As shown in Figure 1C, the peak CR amplitude for control mice is expressed within the expected US period. However, the experimental mice express a weaker, transient response with its peak before US onset.

**Figure 2:**
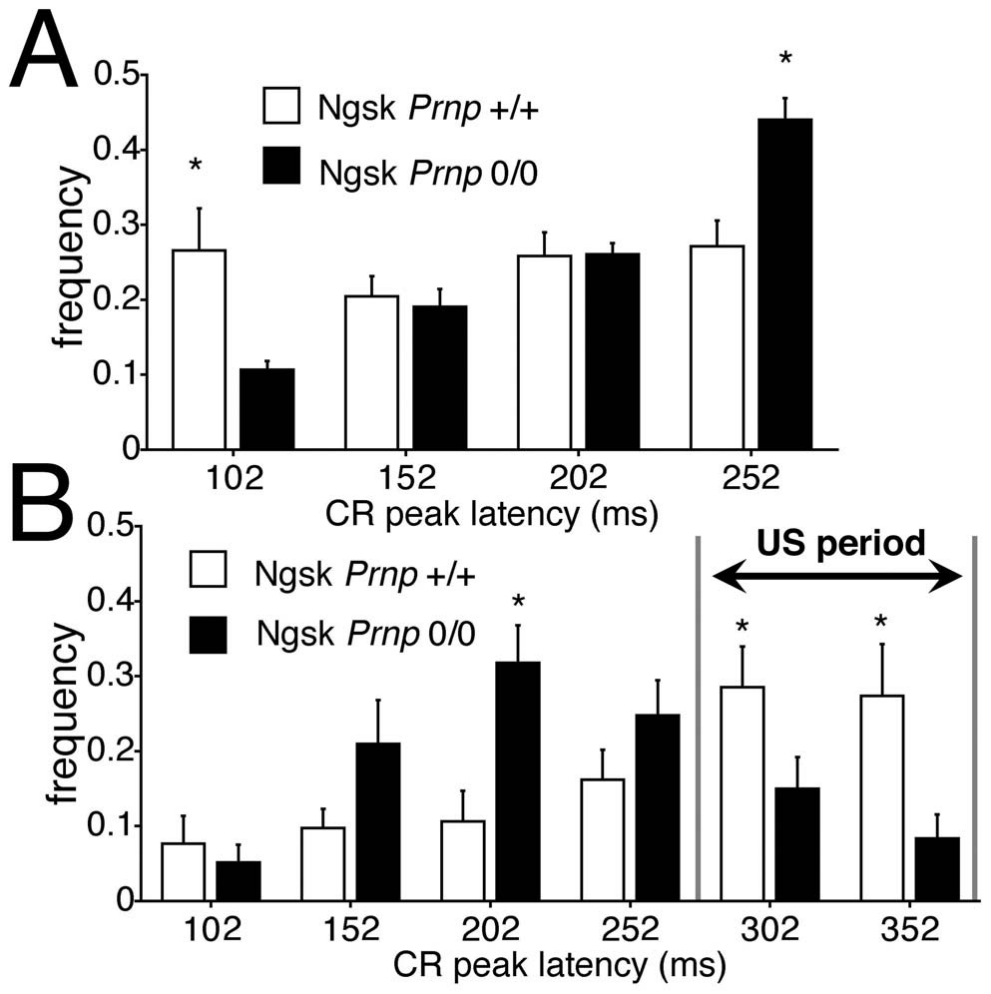
CR timing is altered in 16-week-old Ngsk *Prnp* ^0/0^ mice. (**A**) Histogram showing the normalized frequency of the peak CR plotted as a function of its latency. The CR in 100 trials on day 7 was binned into time windows of 52–102, 102–152, 152–202, and 202–252 ms measured from CS onset in control mice (open column) and Ngsk *Prnp* ^0/0^ mice (experimental mice; closed column). (**B**) Frequency histogram of the CR peak latencies in 10 CS-only trials on day 7 in the time window until the CS end (50-ms widths) for control (open column) and experimental mice (closed column). The adaptive components during US period (252–302 and 302–352 ms from CS onset) of CRs were significantly less in experimental mice. Data points represent the mean ± SEM. *p<0.05.

In addition, we quantitatively analyzed the topography of CR expression in experimental mice at 16 weeks in 2 ways. Figure 2A shows the relative frequency of the CR peak until US onset in 100 trials on day 7, expressed in each time window (52–102, 102–152, 152–202, and 202–252 ms from CS onset). The normalized frequency of CR peak expression in the last time window (202–252 ms) is significantly higher in experimental mice than in control mice (*p*<0.05). Next, the relative frequency of the CR peak on day 7 was re-analyzed using 10 CS-only trials in time windows extended to the US end (52–102, 102–152, 152–202, 202–252, 252–302, and 302–352 ms from CS onset; Figure 2B). Figure 2B shows that the normalized frequency of CR peak expression during the expected US time window (252–302 and 302–352 ms from CS onset) is significantly lower in experimental mice than in control mice (*p*<0.05). Thus, the CR peak latency within the UR period decreased in young Ngsk *Prnp* ^0/0^ mice than in control mice, with a shifted CR peak in experimental mice approximately 100 ms earlier than in control mice.

### CR Acquisition is Severely Impaired in Ngsk *Prnp* ^0/0^ Mice at 40 and 60 Weeks of Age

**Figure 3:**
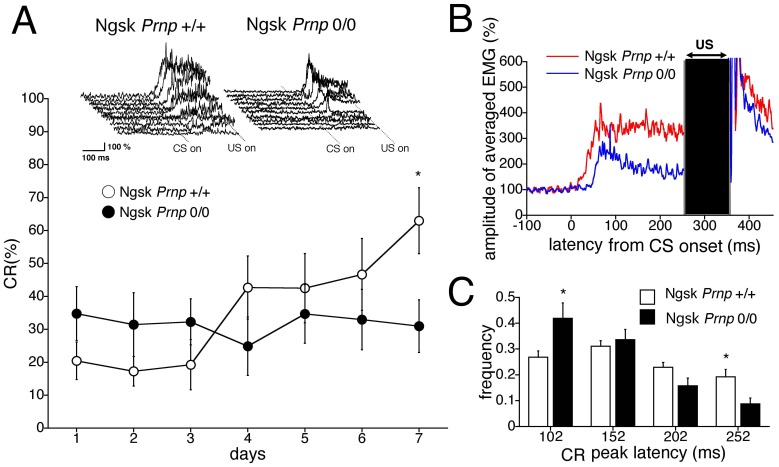
Impaired eyeblink conditioning in 40-week-old Ngsk *Prnp* ^0/0^ mice. (**A**) CR development during a 7-day acquisition session in control (open circle, n = 10) and Ngsk *Prnp* ^0/0^ (experimental mice; closed circle, n = 10) mice aged 40 weeks. The top inset shows individual response topographies of averaged EMGs until US onset in control and experimental mice on day 7. (**B**) Averaged EMG amplitudes during the acquisition session on day 7. All EMG amplitudes obtained in 1 day were summed, representing the average of the eyelid responses. (**C**) Frequency histogram showing the peak CR latencies. The CR on day 7 was binned into time windows of 52–102, 102–152, 152–202, and 202–252 ms measured from CS onset in experimental mice (closed column) and control mice (open column). Data points represent the mean ± SEM. *p<0.05.

Previous studies [13], [15] have reported that 40-week-old Ngsk *Prnp* ^0/0^ mice begin to exhibit histological abnormalities such as PC axonal swelling and demyelination in the spinal cord and peripheral nervous system, but exhibit no behavioral irregularities. However, as shown in Figure 3A, the CR% for the experimental mice at this age was 31.0%±8.0% on the 7th day of training, whereas the CR% for the age-matched control mice progressively increased to 63.2%±9.9% during the 7-day acquisition period. The results of repeated-measures ANOVA revealed significant effects in the interaction between sessions and genotypes (*F*_(6,108)_ = 2.28, *p* = 0.041). The top inset in Figure 3A shows individual EMG topographies averaged by 100 trials on day 7 in both groups. In contrast to 16-week-old mice, these data indicate impaired eyeblink conditioning in the 40-week-old experimental mice. However, they exhibited normal spontaneous eyeblink frequencies comparable to that of the control mice (12.6%±3.2% for control mice and 10.2%±2.3% for experimental mice). Furthermore, the relative number of valid trials (78.8%±8.7% for control mice and 75.8%±7.7% for experimental mice) and startle responses (5.1%±1.9% for control mice and 4.1%±0.9% for experimental mice) did not differ between genotypes. In addition, no differences were detected between control and Ngsk *Prnp* ^0/0^ mice aged 40 weeks in pseudoconditioning or auditory responses (see Figures S1B and S2A). Motor coordination, evaluated using the rota-rod and fixed-bar tests, was still normal in this mutant at this age (data not shown). These results indicate that 40-week-old experimental mice have intact eyeblink motor output and sensitivity to the CS.

Next, we investigated the CR temporal pattern in 40-week-old experimental mice. Figure 3B shows the averaged EMG amplitudes obtained from 40-week-old control and experimental mice on day 7 of the acquisition sessions. The shorter components of the CR peak latency were larger in the experimental mice, whereas all components were comparable in the control mice. Quantitative analyses of the CR temporal properties (Figure 3C) also indicated a significantly higher CR peak expression in the earliest time window (52–102 ms) in experimental mice (*p*<0.05), whereas the relative frequency of CR peak expression in the last time window (202–252 ms) was much smaller than the control mice (*p*<0.05). Thus, CR peak latency was significantly shortened in 40-week-old experimental mice. This aberrant CR timing is more prominent than that observed in 16-week-old experimental mice (Figure 2A and 2B).

**Figure 4:**
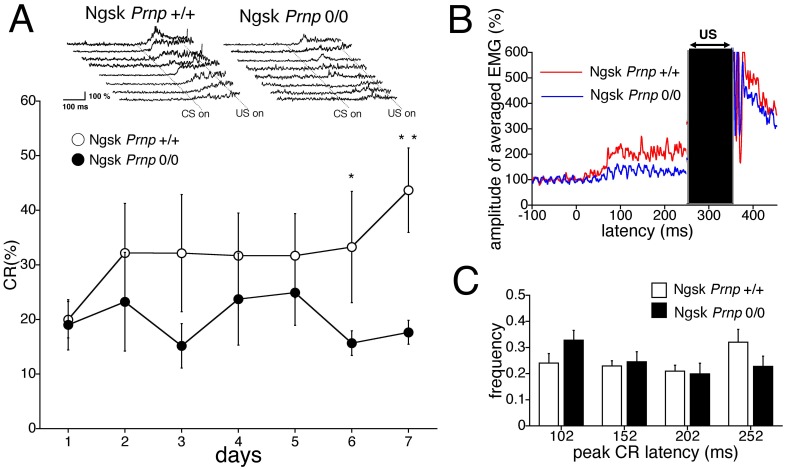
Impaired eyeblink conditioning in 60-week-old Ngsk *Prnp* ^0/0^ mice. (**A**) Eyeblink conditioning performance during the 7-day acquisition training in Ngsk *Prnp* ^0/0^ mice (experimental mice; closed circle, n = 8) and control mice (open circle, n = 8) aged 60 weeks. The top inset shows individual response topographies of averaged EMGs until US onset in control and experimental mice on day 7. (**B**) Averaged EMG amplitudes during the acquisition session on day 7. All EMG amplitudes obtained in 1 day were summed, representing the average of the eyelid responses. (**C**) Frequency histogram showing the peak CR latencies. The CR on day 7 was binned into time windows of 52–102, 102–152, 152–202, and 202–252 ms measured from CS onset in experimental mice (closed column) and control mice (open column). Data points represent the mean ± SEM. Data points represent the mean ± SEM. *p<0.05, **p<0.01.

Next, we investigated eyeblink conditioning in the 60-week-old Ngsk *Prnp* ^0/0^ mice. Sixty-week-old Ngsk *Prnp* ^0/0^ (experimental) mice began to exhibit dramatic loss of PCs throughout the cerebellar vermis [13], [15]. Figure 4A shows the time course of the CR% in 60-week-old controls and experimental mice. The CR% for the wild-type mice gradually increased to 42.9%±8.2% on day 7. This comparatively low value is likely due to normal aging effects [58], although ANOVA revealed no statistically significant difference in the CR performance between 16- and 60-week-old control mice (statistical data not shown). However, CR acquisition was profoundly impaired in experimental mice, even when the age-related impairment was taken into account. The CR% was only 17.6%±2.2% on the 7th day of training. Although found no significant effects in the interaction between the sessions and genotypes (*F*_(6, 84)_ = 0.46, *p* = 0.86), we did find significant effects due to genotype (*F*_(1,14)_ = 6.93, *p = *0.019). The top inset in Figure 4A shows individual EMG topographies averaged by 100 trials on day 7 in both groups. Figure 4B depicts the averaged EMG amplitudes obtained from 60-week-old control and experimental mice on day 7. These analyses clearly indicated lower amplitude of the CR component in the experimental mice than in the control mice. Analyses of the temporal pattern of CR expression failed to detect any significant difference between the control and experimental mice, although a similar tendency of shortened peak CR latency was observed in the experimental mice (Figure 4C).

Similar to the 16- and 40-week-old mice, the 60-week-old mice showed no difference in spontaneous eyeblink frequency between the 2 genotypes (10.5%±2.9% for control mice and 10.2%±2.1% for Ngsk *Prnp* ^0/0^ mice). The relative number of valid trials (74.2%±6.8% for control mice and 71.6%±9.6% for experimental mice) and startle responses (3.7%±0.7% for control mice and 5.1%±1.1% for experimental mice) were also not statistically different between the genotypes. Furthermore, no differences were found between control groups and experimental mice aged 60 weeks in pseudoconditioning (see Figure S1C) or the baseline of eyeblink frequencies in the presence of tone CS (see Figure S1C and S2A), although considerable motor incoordination was observed at this age (data not shown). These results imply that eyeblink motor output and sensitivity to the CS remain intact in Ngsk *Prnp* ^0/0^ mice even at the age of 60 weeks.

### Altered Timing but Normal CR Acquisition in Young ZrchI *Prnp* ^0/0^ Mice

**Figure 5:**
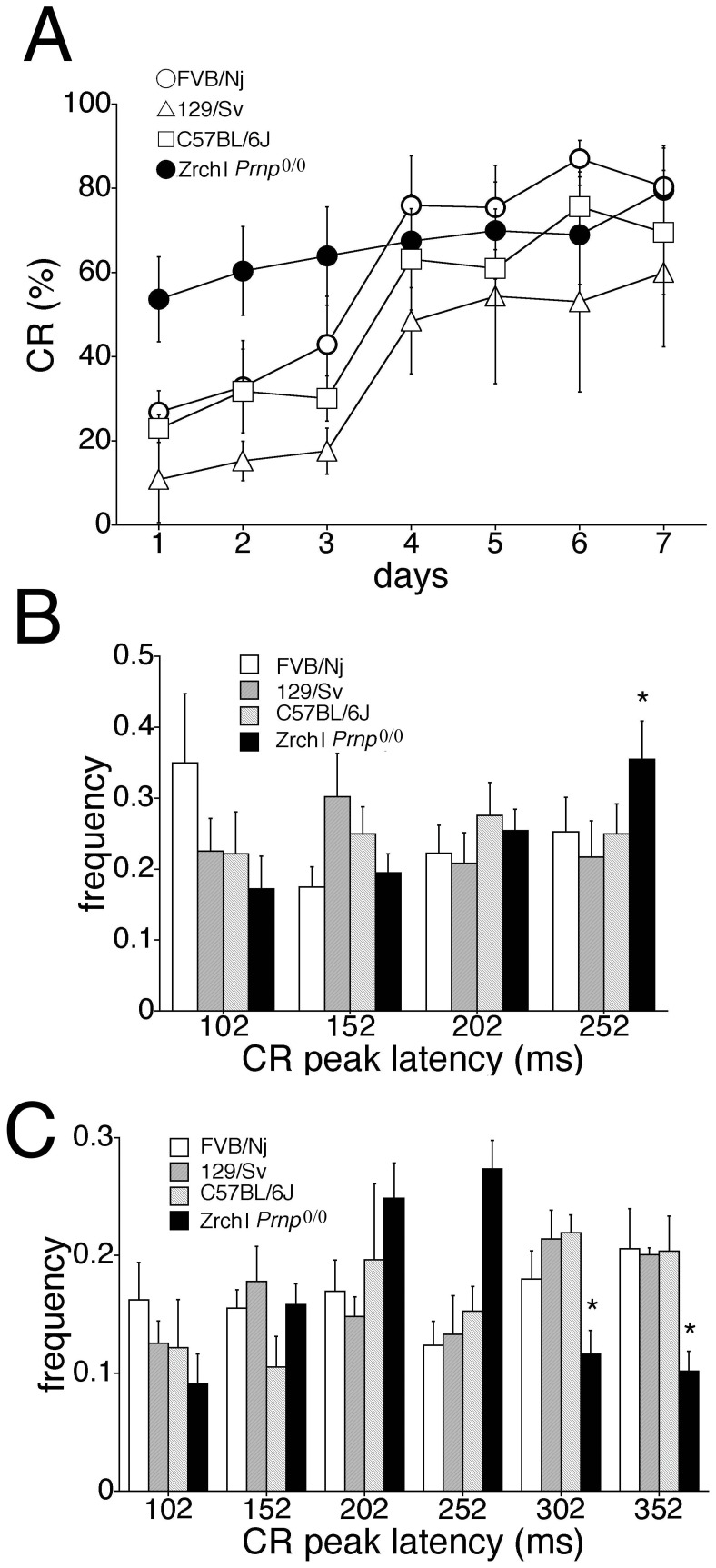
Eyeblink conditioning in ZrchI *Prnp* ^0/0^ mice. (**A**) Development of the CR during 7-day acquisition training in ZrchI *Prnp* ^0/0^ mice (closed circle, n = 9), FVB/Nj mice (open circle, n = 7), 129/Sv mice (open triangle, n = 5), and C57BL/6J mice (open square, n = 6) aged 18–22 weeks. (**B**) Histogram showing the normalized frequency of the peak CR plotted as a function of its latency. Histograms showing the frequency of the CR plotted as a function of its latency. The CR in 100 trials on day 7 was binned into time windows of 52–102, 102–152, 152–202, and 202–252 ms measured from CS onset in the three control strains and ZrchI *Prnp* ^0/0^ mice (closed column). (**C**) Frequency histogram of the CR peak latencies in 10 CS-only trials on day 7 in extended time windows (52–102, 102–152, 152–202, 202–252, 252–302, and 302–352 ms from CS onset) for the three control strains and ZrchI *Prnp* ^0/0^ mice (closed column). Data points represent the mean ± SEM. *p<0.05.

We next investigated whether these behavioral abnormalities in Ngsk *Prnp* ^0/0^ mice were also observed in another line of PrP^C^-deficient mice, ZrchI *Prnp* ^0/0^ mice, which show neither neurodegeneration nor PrPLP/Dpl ectopic expression [8], [59]. Figure 5A shows the CR% during the 7-day acquisition session in ZrchI *Prnp* ^0/0^ (experimental) mice and 3 wild-type control strains (C57BL/6J, 129/SvJ, and FVB/Nj) at 18–22 weeks of age. Although eyeblink conditioning in mice depends on the genetic background [60], we observed no significant difference in CR% among the 3 control strains. Figure 5A shows development of the CR%. Experimental mice, as well as young Ngsk *Prnp* ^0/0^ mice (Figure 1A), seemed to exhibit faster CR acquisition, although the results of an ANOVA revealed no sessions and genotype interaction effect (*p* = 0.152) and no genotype effect (*p* = 0.103).

The relative frequency of the CR peak in 100 trials in each time window (52–102, 102–152, 152–202, and 202–252 ms from CS onset) on day 7 (Figure 5B) demonstrated that the last component of CR expression in 202–252 ms was significantly higher in experimental mice than in the 3 control groups (*p*<0.05). However, the relative frequency of the CR peak in 10 CS-only trials in each time window (52–102, 102–152, 152–202, 202–252, 252–302, and 302–352 ms from CS onset) on day 7 (Figure 5C) indicated that the normalized frequency of CR peak expression during the US time window (252–302 and 302–352 ms from CS onset) was significantly lower in the experimental mice than in the 3 control strains (*p*<0.01). Thus, the shift of CR peak latency to approximately 100 ms earlier was common to the 2 lines of *Prnp* ^0/0^ mice. We found no differences between control groups and experimental mice in spontaneous eyeblink frequency (12.4±2.7% for control mice and 16.1±3.5% for experimental mice), startle response frequency (3.1±0.7% for control mice and 4.6±0.7% for experimental mice), pseudoconditioning (See Figure S1D), or eyeblink frequencies in the presence of CS (See Figure S2B). Motor coordination, evaluated by rota-rod and fixed-bar tests, was normal in the experimental mice (data not shown).

## Discussion

In the present study, we investigated cerebellum-dependent eyeblink conditioning in PrP^C^-deficient Ngsk *Prnp* ^0/0^ mice. Eyeblink conditioning has been used as an evaluation system for human dementias, including Alzheimer’s disease [56], [57], [61]–[63] and aging-related learning deficit [58], [62], [64], [65]. Therefore, it represents a useful system for evaluating the learning ability of mutant animals and elucidating the mechanisms underlying human neurodegenerative diseases. This initially prompted us to examine eyeblink conditioning in Ngsk *Prnp* ^0/0^ mice, which exhibit late-onset PC degeneration, and are an animal model of progressive cerebellar degeneration disorder. Our results revealed that Ngsk *Prnp* ^0/0^ mice exhibit significant CR incidence deficits in eyeblink conditioning as early as at 40 weeks of age. The impairment became more pronounced in 60-week-old Ngsk *Prnp* ^0/0^ mice, indicating an age-dependent deterioration of CR acquisition in eyeblink conditioning. We also analyzed the learning-dependent timing of eyeblink CRs. The CR timing was altered in advanced aged Ngsk *Prnp* ^0/0^ mice as well as, unexpectedly, in 16-week-old Ngsk *Prnp* ^0/0^ mice, before any apparent neurological abnormalities emerged. The altered CR timing was also observed in another line of PrP^C^ KO mice, ZrchI *Prnp* ^0/0^ mice. These results suggest that PrP^c^ is involved in cerebellar function for CR timing during eyeblink conditioning.

### Impaired Acquisition of Conditioned Eyeblink Response in Aged Ngsk *Prnp* ^0/0^ Mice

Sixteen-week-old Ngsk *Prnp* ^0/0^ mice, which show no apparent behavioral or histological abnormalities [13], exhibited intact CR acquisition (Figure 1A). ZrchI *Prnp* ^0/0^ mice aged 18–22 weeks also exhibited normal CR incidence during the acquisition secession (Figure 5A). However, 40-week-old and 60-week-old Ngsk *Prnp* ^0/0^ mice showed significant CR acquisition impairment (Figures 3 and 4). It should be noted that the control mice aged 60 weeks also exhibited a deterioration tendency related to normal aging effects [58]. Because histological changes of the CNS (including abnormal myelination in the spinal cord) occur at approximately 40 weeks of age [15], it is most likely that impaired CR acquisition is due to these neurological abnormalities, particularly cerebellar PC loss [24], [28], [66], [67]. This possibility could be supported by previous results obtained using *pcd* (Purkinje cell degenerated) mutant mice [68], in which the postnatal death of virtually all cerebellar PCs occurred during the third and fourth postnatal week [69]. The *pcd* mutant mice exhibited severely impaired CR acquisition during delay eyeblink conditioning, indicating the necessity of an intact cerebellar cortex, particularly preserved cerebellar PCs, for acquisition in delay eyeblink conditioning. In addition, experiments using globally depleted cerebellar PCs with the immunotoxin OX7-saporin and investigations of the individual differences of PCs have shown that the degree of PC loss was well correlated with the magnitude of CR acquisition impairments in rats or rabbits [70], [71].

Furthermore, patients with cerebellar neurodegenerative diseases are reported to exhibit severe impairment of CR incidence during delay eyeblink conditioning [72]–[76]. Our results support the notion that delay eyeblink conditioning is highly sensitive for detecting cerebellar PCs neurodegenerative changes and deficits in both humans and rodents. However, in the present study, we did not examine delay eyeblink conditioning in aged Zrch *Prnp* ^0/0^ mice (at the ages of 40 and 60 weeks). Therefore, we cannot deny the possibility that other undiscovered, *Prnp* deficiency-induced, age-dependent abnormalities might have impaired eyeblink conditioning in aged Ngsk *prnp* ^0/0^ mice.

We found no difference between controls and *Prnp* ^0/0^ mice in spontaneous eyeblink frequency, sensitivity to the tone, and electrical shock (data not shown). The auditory responses to tone CS were not altered in Ngsk and Zrch *Prnp* ^0/0^ mice during the age periods examined, despite the trend of higher eyeblink frequencies in the mutants at 16 and 40 weeks of age (Figure S2 and Text S1). Pseudoconditioning was also not altered in *Prnp* ^0/0^ mice (Figure S1 and Text S1). These data confirm that the impairments in older Ngsk *Prnp* ^0/0^ mice did not originate from basic performance disturbances (including sensitivity to the US and CS). Nevertheless, Ngsk *Prnp* ^0/0^ mice aged 60 weeks exhibited considerable ataxia and motor dysfunction [13]; therefore, at this age, we cannot exclude the possibility that the eyeblink CR deficit was due to general ataxia rather than a specific learning deficit in Ngsk *Prnp* ^0/0^ mice. However, several examples of intact eyeblink conditioning despite severe ataxia have been reported in previous studies on knockout mice [77], [78]. Thus, eyeblink conditioning deficits and motor function deficits may not necessarily be closely correlated. We observed apparently higher CR acquisition in 16- and 40- week-old Ngsk and Zrch *Prnp* ^0/0^ mice at the early phase of delay eyeblink conditioning (Figures 1A, 3A, and 5A). We do not deny that the hyperactivity might be due to higher base line of eyeblink frequencies under tone CS in the *Prnp* ^0/0^ mice (Figure S2). However, even if this is the case, the important point is that 40-week-old Ngsk *Prnp* ^0/0^ mice exhibited lower CR% in the attained level, despite the higher eyeblink frequency baseline.

Recently, Zrch *Prnp* ^0/0^ mice were reported to have more excitability as well as larger and longer long-term potentiation (LTP) at the hippocampal CA1 synapse *in vivo* than their littermate controls [79]. The hippocampus is also important for simple delay eyeblink conditioning [80]–[82]; that is, the hippocampus affects the rate of CR acquisition during the delay conditioning [83]. Indeed, we previously showed that mutant mice lacking the NMDA receptor ε1 subunit, which have impaired hippocampal CA1-LTP, exhibited slower CR acquisition but maintained an intact attained level of CRs during a 7-day acquisition session on delay eyeblink conditioning [84], [85]. Taken together, our results suggest that the rapid acquisition, but normal attained level, of CRs during delay eyeblink conditioning observed in young Ngsk and Zrch *Prnp* ^0/0^ mice (Figures 1A and 5A) might be explained by the larger hippocampal LTP in these strains.

### Altered Timing of Conditioned Eyeblink Response in Ngsk and ZrchI *Prnp* ^0/0^ Mice

In eyeblink conditioning, the CR is viewed as a prediction of the imminence of the US, and the peak CR amplitude coincides with the timing of the US. The results of lesion experiments have suggested the involvement of the cerebellar cortical circuit in CR timing [53], although it has been recently indicated that electrotonic coupling among olivary neurons by gap junctions is important for the proper timing of eyelid CRs [86]. Previous theoretical and simulation studies have suggested that the interactions between populations of granule cells and Golgi cells are engaged in the regulation of CR timing [49], [52], [87], [88]. We have shown that young Ngsk *Prnp* ^0/0^ mice exhibit altered CR timing (Figure 2A and 2B). ZrchI *Prnp* ^0/0^ mice aged 18–22 weeks, which also exhibit no histological abnormalities [8], [10], reproducibly exhibited altered timing of the CR (Figure 5B and 5C); these mutant mice had apparently longer CR peak latencies than control mice, as evident in the results of a conventional latency analysis (Figures 2A and 5B). However, taking into account of the CR components during US period, CR peaks of these mutant mice were substantially shifted earlier than those of control mice (Figures 2B and 5C). This temporal pattern resembles that observed in *waggler* and *stargazer* mutant mice, which have dysfunctional cerebellar granule cells and deficits in brain-derived neurotrophic factor [89]–[92]. In addition, intracellular calcium homeostasis was disturbed in cultured cerebellar granular cells from ZrchI *Prnp* ^0/0^ mice [93]. This is also similar to the phenotype of *waggler* mutant mice, in which a putative neuronal Ca^2+^ channel γ subunit is disrupted in cerebellar granule cells. Thus, some abnormal granule cell signaling cascades could affect CR timing in both young Ngsk and ZrchI *Prnp* ^0/0^ mice. Indeed, previous immunohistochemical studies have suggested that PrP^C^ is highly expressed in the axon terminals of cerebellar granule cells [94]. Furthermore, ZrchI *Prnp* ^0/0^ mice exhibited abnormal granule cell excitability and altered synaptic plasticity at synapses between mossy fibers and granule cells [95]. Therefore, examination of the neurophysiological properties of granule cells from Ngsk *Prnp* ^0/0^ mice would be important. Recently, the Delgado-García group proposed a reinforcing-modulating role of posterior interpositus neurons in the proper performance of eyeblink CRs [42], [43]. Thus, it would be interesting to investigate neurophysiological properties from the interpositus neurons of *Prnp* ^0/0^ mice because PrP^C^ is highly expressed in cerebellar deep nuclei [46].

On the other hand, in Ngsk *Prnp* ^0/0^ mice aged 40 weeks, the early components of the CR occurring immediately after CS onset (52–102 ms) dominate, whereas later components (202–252 ms) of the CR are much decreased even by conventional timing analyses (Figure 3C). Ngsk *Prnp* ^0/0^ mice aged 60 weeks exhibited a similar tendency, i.e., shortened CR peak timing, although no significant differences were found in any component between Ngsk *Prnp* ^0/0^ and control mice (Figure 4C). We consider that analyses of the temporal pattern of CR expression were not applicable in Ngsk *Prnp* ^0/0^ mice aged 60 weeks, because only a few CRs were observed in the 60-week-old Ngsk *Prnp* ^0/0^ mice. This shortened aberrant CR timing pattern observed in 40-week-old and 60-week-old Ngsk *Prnp* ^0/0^ mice is similar to that observed in animals with a lesioned cerebellar cortex [53] and in *pcd* mutant mice, which lose nearly all of their PCs during development [68]. Additionally, a shortened CR peak timing was also observed in Lurcher mutant mice, which lack PCs and granule cells in the cerebellar cortex due to a mutation in the GluRδ2 receptor [96]. Furthermore, patients with cortical cerebellar degeneration also show similar timing deficits of eyeblink CRs, suggesting that some areas of the superior cerebellar cortex are important for the formation of appropriately timed conditioned eyeblink responses in humans [51], [76]. Thus, the shorter latency of the CR in 60-week-old Ngsk *Prnp* ^0/0^ mice might also be explained by cerebellar cortical degeneration. In wild-type mice, the short-latency responses are not expressed, possibly because interpositus nuclei are strongly inhibited by PC activity during the early CS phases, whereas in older Ngsk *Prnp* ^0/0^ mice, the short-latency response pathway is unmasked because of the loss of cerebellar cortex output [52].

### Conclusions

In this cross-sectional study, we investigated eyeblink conditioning in Ngsk *Prnp* ^0/0^ mice, which exhibit cerebellar neurodegenerative symptoms late in life. We found age-dependent alterations of eyeblink conditioning in 2 indices: (i) CR incidence and (ii) the timing of CR expression. The CR incidence impairment in older Ngsk *Prnp* ^0/0^ mice could be attributed to progressive degeneration of PCs, indicating the importance of the cerebellar cortex in acquisition of eyeblink conditioning. Intriguingly, the CR timing was altered in both young Ngsk *Prnp* ^0/0^ mice and older Ngsk *Prnp* ^0/0^ mice. The shortened latency observed in young and older Ngsk *Prnp* ^0/0^ mice is consistent with the CR temporal pattern observed in cerebellar cortex-lesioned animals [53], [68], [96] and cerebellar patients [51], [76], suggesting that cerebellar degeneration affects aberrant CR timing. Finally, the altered CR timing was also observed in young ZrchI *Prnp* ^0/0^ mice, suggesting that PrP^C^ is involved in cerebellar function for CR timing during eyeblink conditioning.

## Supporting Information

Figure S1 **Pseudoconditioning in prion knockout mice.** (**A**) Pseudoconditioning in Ngsk *prnp* ^0/0^ (n = 4) and their control (n = 4) mice at 16 weeks old. (**B**) Pseudoconditioning in Ngsk *prnp* ^0/0^ (n = 8) and their control (n = 9) mice at 40 weeks old. (**C**) Pseudoconditioning in Ngsk *prnp* ^0/0^ (n = 8) and their control (n = 8) mice at 60 weeks old. (**D**) Pseudoconditioning in Zrch *prnp* ^0/0^ (n = 10) and their control (n = 9) mice at 60 weeks old. The data points represent the mean ± SEM.
(TIF)

Figure S2 **Normal auditory response to tone CS in prion knockout mice.** (**A**) Frequency of eyeblink response during CS in Ngsk *prnp* ^0/0^ (KO) and their control (CT) mice at the ages of 16, 40, and 60 weeks. (**B**) Frequency of eyeblink response during CS in Zrch *prnp* ^0/0^ (KO) and their control mice (CT). The data points represent the mean ± SEM. The values in parentheses above the column indicate the number of mice used.
(TIF)

Text S1 **Supplemental Methods and Supplemental Results.**
(DOCX)

## References

[1] Collinge J (1999) Variant Creutzfeldt-Jakob disease. Lancet 354: 317–323. 10440324 10.1016/S0140-6736(99)05128-4

[2] Gajdusek DC (1977) Unconventional viruses and the origin and disappearance of kuru. Science 197: 943–960. 142303 10.1126/science.142303

[3] Horwich AL, Weissman JS (1997) Deadly conformations–protein misfolding in prion disease. Cell 89: 499–510. 9160742 10.1016/s0092-8674(00)80232-9

[4] Prusiner SB (1997) Prion diseases and the BSE crisis. Science 278: 245–251. 9323196 10.1126/science.278.5336.245

[5] Prusiner SB (1998) Prions. Proc Natl Acad Sci USA 95: 13363–13383. 9811807 10.1073/pnas.95.23.13363 PMC33918

[6] Masullo C, Salvatore M, Macchi G, Genuardi M, Pocchiari M (1994) Progressive dementia in a young patient with a homozygous deletion of the PrP gene. Ann N Y Acad Sci 724: 358–360. 8030960 10.1111/j.1749-6632.1994.tb38931.x

[7] Schulz-Schaeffer WJ, Giese A, Windl O, Kretzschmar HA (1996) Polymorphism at codon 129 of the prion protein gene determines cerebellar pathology in Creutzfeldt-Jakob disease. Clin Neuropathol 15: 353–357. 8937783

[8] Bueler H, Fischer M, Lang Y, Bluethmann H, Lipp HP, et al (1992) Normal development and behaviour of mice lacking the neuronal cell-surface PrP protein. Nature 356: 577–582. 1373228 10.1038/356577a0

[9] Kuwahara C, Takeuchi AM, Nishimura T, Haraguchi K, Kubosaki A, et al (1999) Prions prevent neuronal cell-line death. Nature 400: 225–226. 10421360 10.1038/22241

[10] Manson JC, Clarke AR, Hooper ML, Aitchison L, McConnell I, et al (1994) 129/Ola mice carrying a null mutation in PrP that abolishes mRNA production are developmentally normal. Mol Neurobiol 8: 121–127. 7999308 10.1007/BF02780662

[11] Moore RC, Lee IY, Silverman GL, Harrison PM, Strome R, et al (1999) Ataxia in prion protein (PrP)-deficient mice is associated with upregulation of the novel PrP-like protein doppel. J Mol Biol 292: 797–817. 10525406 10.1006/jmbi.1999.3108

[12] Sakaguchi S, Katamine S, Shigematsu K, Nakatani A, Moriuchi R, et al (1995) Accumulation of proteinase K-resistant prion protein (PrP) is restricted by the expression level of normal PrP in mice inoculated with a mouse-adapted strain of the Creutzfeldt-Jakob disease agent. J Virol 69: 7586–7592. 7494265 10.1128/jvi.69.12.7586-7592.1995 PMC189697

[13] Sakaguchi S, Katamine S, Nishida N, Moriuchi R, Shigematsu K, et al (1996) Loss of cerebellar Purkinje cells in aged mice homozygous for a disrupted PrP gene. Nature 380: 528–531. 8606772 10.1038/380528a0

[14] Nishida N, Katamine S, Shigematsu K, Nakatani A, Sakamoto N, et al (1997) Prion protein is necessary for latent learning and long-term memory retention. Cell Mol Neurobiol 17: 537–545. 9353594 10.1023/A:1026315006619 PMC11560205

[15] Nishida N, Tremblay P, Sugimoto T, Shigematsu K, Shirabe S, et al (1999) A mouse prion protein transgene rescues mice deficient for the prion protein gene from purkinje cell degeneration and demyelination. Lab Invest 79: 689–697. 10378511

[16] Li A, Sakaguchi S, Atarashi R, Roy BC, Nakaoke R, et al (2000) Identification of a novel gene encoding a PrP-like protein expressed as chimeric transcripts fused to PrP exon 1/2 in ataxic mouse line with a disrupted PrP gene. Cell Mol Neurobiol 20: 553–567. 10930132 10.1023/A:1007059827541 PMC11537530

[17] Westaway D, Daude N, Wohlgemuth S, Harrison P (2011) The PrP-like proteins Shadoo and Doppel. Top Curr Chem 305: 225–256. 21728138 10.1007/128_2011_190

[18] Moore RC, Mastrangelo P, Bouzamondo E, Heinrich C, Legname G, et al (2001) Doppel-induced cerebellar degeneration in transgenic mice. Proc Natl Acad Sci USA 98: 15288–15293. 11734625 10.1073/pnas.251550798 PMC65022

[19] Li A, Sakaguchi S, Shigematsu K, Atarashi R, Roy BC, et al (2000) Physiological expression of the gene for PrP-like protein, PrPLP/Dpl, by brain endothelial cells and its ectopic expression in neurons of PrP-deficient mice ataxic due to Purkinje cell degeneration. Am J Pathol 157: 1447–1452. 11073804 10.1016/S0002-9440(10)64782-7 PMC1885740

[20] Mo H, Moore RC, Cohen FE, Westaway D, Prusiner SB, et al (2001) Two different neurodegenerative diseases caused by proteins with similar structures. Proc Natl Acad Sci USA 98: 2352–2357. 11226243 10.1073/pnas.051627998 PMC30142

[21] Behrens A, Genoud N, Naumann H, Rulicke T, Janett F, et al (2002) Absence of the prion protein homologue Doppel causes male sterility. EMBO J 21: 3652–3658. 12110578 10.1093/emboj/cdf386 PMC125402

[22] Legname G, Nelken P, Guan Z, Kanyo ZF, DeArmond SJ, et al (2002) Prion and doppel proteins bind to granule cells of the cerebellum. Proc Natl Acad Sci USA 99: 16285–16290. 12446843 10.1073/pnas.242611999 PMC138603

[23] Peoc’h K, Serres C, Frobert Y, Martin C, Lehmann S, et al (2002) The human “prion-like” protein Doppel is expressed in both Sertoli cells and spermatozoa. J Biol Chem 277: 43071–43078. 12200435 10.1074/jbc.M206357200

[24] Sakurai-Yamashita Y, Sakaguchi S, Yoshikawa D, Okimura N, Masuda Y, et al (2005) Female-specific neuroprotection against transient brain ischemia observed in mice devoid of prion protein is abolished by ectopic expression of prion protein-like protein. Neuroscience 136: 281–287. 16198494 10.1016/j.neuroscience.2005.06.095

[25] Silverman GL, Qin K, Moore RC, Yang Y, Mastrangelo P, et al (2000) Doppel is an N-glycosylated, glycosylphosphatidylinositol-anchored protein. Expression in testis and ectopic production in the brains of *Prnp* ^0/0^ mice predisposed to Purkinje cell loss. J Biol Chem 275: 26834–26841. 10842180 10.1074/jbc.M003888200

[26] Yoshikawa D, Kopacek J, Yamaguchi N, Ishibashi D, Yamanaka H, et al (2007) Newly established in vitro system with fluorescent proteins shows that abnormal expression of downstream prion protein-like protein in mice is probably due to functional disconnection between splicing and 3′ formation of prion protein pre-mRNA. Gene 386: 139–146. 17034959 10.1016/j.gene.2006.08.028

[27] Kopacek J, Sakaguchi S, Shigematsu K, Nishida N, Atarashi R, et al (2000) Upregulation of the genes encoding lysosomal hydrolases, a perforin-like protein, and peroxidases in the brains of mice affected with an experimental prion disease. J Virol 74: 411–417. 10590130 10.1128/jvi.74.1.411-417.2000 PMC111552

[28] Shmerling D, Hegyi I, Fischer M, Blattler T, Brandner S, et al (1998) Expression of amino-terminally truncated PrP in the mouse leading to ataxia and specific cerebellar lesions. Cell 93: 203–214. 9568713 10.1016/s0092-8674(00)81572-x

[29] Weissmann C, Aguzzi A (1999) Perspectives: neurobiology. PrP’s double causes trouble. Science 286: 914–915. 10577243 10.1126/science.286.5441.914

[30] Kim JJ, Thompson RF (1997) Cerebellar circuits and synaptic mechanisms involved in classical eyeblink conditioning. Trends Neurosci 20: 177–181. 9106359 10.1016/s0166-2236(96)10081-3

[31] Thompson RF, Kim JJ (1996) Memory systems in the brain and localization of a memory. Proc Natl Acad Sci USA 93: 13438–13444. 8942954 10.1073/pnas.93.24.13438 PMC33628

[32] Thompson RF, Krupa DJ (1994) Organization of memory traces in the mammalian brain. Annu Rev Neurosci 17: 519–549. 8210186 10.1146/annurev.ne.17.030194.002511

[33] Attwell PJ, Cooke SF, Yeo CH (2002) Cerebellar function in consolidation of a motor memory. Neuron 34: 1011–1020. 12086647 10.1016/s0896-6273(02)00719-5

[34] Bracha V (2004) Role of the cerebellum in eyeblink conditioning. Prog Brain Res 143: 331–339. 14653177 10.1016/S0079-6123(03)43032-X

[35] Krupa DJ, Thompson JK, Thompson RF (1993) Localization of a memory trace in the mammalian brain. Science 1260: 989–991. 10.1126/science.8493536 8493536

[36] Mauk MD (1997) Roles of cerebellar cortex and nuclei in motor learning: contradictions or clues? Neuron 18: 343–346. 9115728 10.1016/s0896-6273(00)81235-0

[37] Raymond JL, Lisberger SG, Mauk MD (1996) The cerebellum: a neuronal learning machine? Science 272: 1126–1131. 8638157 10.1126/science.272.5265.1126

[38] Yeo CH, Hesslow G (1998) Cerebellum and conditioned reflexes. Trends Cogn Sci 2: 322–330. 21227228 10.1016/s1364-6613(98)01219-4

[39] Jiménez-Díaz L, Navarro-López J de D, Gruart A, Delgado-García JM (2004) Role of cerebellar interpositus nucleus in the genesis and control of reflex and conditioned eyelid responses. J Neurosci 24: 9138–9145. 15483132 10.1523/JNEUROSCI.2025-04.2004 PMC6730068

[40] McCormick DA, Thompson RF (1984) Cerebellum: essential involvement in the classically conditioned eyelid response. Science 223: 296–299. 6701513 10.1126/science.6701513

[41] Porras-García E (2010) Sánchez-Campusano R, Martinez-Vargas D, Domínguez-del-Toro E, Cendelín J, et al (2010) Behavioral characteristics, associative learning capabilities, and dynamic association mapping in an animal model of cerebellar degeneration. J Neurophysiol 104: 346–365. 20410355 10.1152/jn.00180.2010

[42] Sánchez-Campusano R, Gruart A, Delgado-García JM (2007) The cerebellar interpositus nucleus and the dynamic control of learned motor responses. J Neurosci 27: 6620–6632. 17581949 10.1523/JNEUROSCI.0488-07.2007 PMC6672710

[43] Sánchez-Campusano R, Gruart A, Delgado-García JM (2009) Dynamic associations in the cerebellar-motoneuron network during motor learning. J Neurosci 29: 10750–10763. 19710326 10.1523/JNEUROSCI.2178-09.2009 PMC6665687

[44] Welsh JP, Harvey JA (1989) Cerebellar lesions and the nictitating membrane reflex: performance deficits of the conditioned and unconditioned response. J Neurosci 9: 299–311. 2913208 10.1523/JNEUROSCI.09-01-00299.1989 PMC6569988

[45] Yeo CH, Hardiman MJ, Glickstein M (1985) Classical conditioning of the nictitating membrane response of the rabbit. II. Lesions of the cerebellar cortex. Exp Brain Res 60: 99–113. 4043286 10.1007/BF00237023

[46] Laine J, Marc ME, Sy MS, Axelrad H (2001) Cellular and subcellular morphological localization of normal prion protein in rodent cerebellum. Eur J Neurosci 14: 47–56. 11488948 10.1046/j.0953-816x.2001.01621.x

[47] Salès N, Rodolfo K, Hassig R, Faucheux B, Di Giamberardino L, et al (1998) Cellular prion protein localization in rodent and primate brain. Eur J Neurosci 10: 2464–2471. 9749773 10.1046/j.1460-9568.1998.00258.x

[48] Tanji K, Saeki K, Matsumoto Y, Takeda M, Hirasawa K, et al (1995) Analysis of PrPc mRNA by in situ hybridization in brain, placenta, uterus and testis of rats. Intervirology 38: 309–315. 8880380 10.1159/000150457

[49] Buonomano DV, Mauk MD (1994) Neural network model of the cerebellum: Temporal discrimination and the timing of motor responses. Neural Comp 6: 38–55.

[50] Garcia KS, Mauk MD (1998) Pharmacological analysis of cerebellar contributions to the timing and expression of conditioned eyelid responses. Neuropharmacology 37: 471–480. 9704988 10.1016/s0028-3908(98)00055-0

[51] Gerwig M, Hajjar K, Dimitrova A, Maschke M, Kolb FP, et al (2005) Timing of conditioned eyeblink responses is impaired in cerebellar patients. J Neurosci 25: 3919–3931. 15829644 10.1523/JNEUROSCI.0266-05.2005 PMC6724917

[52] Medina JF, Garcia KS, Nores WL, Taylor NM, Mauk MD (2000) Timing mechanisms in the cerebellum: testing predictions of a large-scale computer simulation. J Neurosci 20: 5516–5525. 10884335 10.1523/JNEUROSCI.20-14-05516.2000 PMC6772322

[53] Perrett SP, Ruiz BP, Mauk MD (1993) Cerebellar cortex lesions disrupt learning-dependent timing of conditioned eyelid responses. J Neurosci 13: 1708–1718. 8463846 10.1523/JNEUROSCI.13-04-01708.1993 PMC6576722

[54] Kishimoto Y, Kano M (2006) Endogenous cannabinoid signaling through the CB1 receptor is essential for cerebellum-dependent discrete motor learning. J Neurosci 26: 8829–8837. 16928872 10.1523/JNEUROSCI.1236-06.2006 PMC6674369

[55] Kishimoto Y, Fujimichi R, Araishi K, Kawahara S, Kano M, et al (2002) mGluR1 in cerebellar Purkinje cells is required for normal association of temporally contiguous stimuli in classical conditioning. Eur J Neurosci 16: 2416–2424. 12492436 10.1046/j.1460-9568.2002.02407.x

[56] Kishimoto Y, Oku I, Nishigawa A, Nishimoto A, Kirino Y (2012) Impaired long-trace eyeblink conditioning in a Tg2576 mouse model of Alzheimer’s disease. Neurosci Lett 506: 155–159. 22085694 10.1016/j.neulet.2011.10.071

[57] Kishimoto Y, Kirino Y (2013) Presenilin 2 mutation accelerates the onset of impairment in trace eyeblink conditioning in a mouse model of Alzheimer’s disease overexpressing human mutant amyloid precursor protein. Neurosci Lett In press. doi: 10.1016/j.neulet.2013.01.025. 10.1016/j.neulet.2013.01.025 23370287

[58] Kishimoto Y, Suzuki M, Kawahara S, Kirino Y (2001) Age-dependent impairment of delay and trace eyeblink conditioning in mice. Neuroreport 12: 3349–3352. 11711884 10.1097/00001756-200110290-00040

[59] Weissmann C, Bueler H, Fischer M, Sailer A, Aguzzi A, et al (1994) PrP-deficient mice are resistant to scrapie. Ann N Y Acad Sci 724: 235–240. 8030944 10.1111/j.1749-6632.1994.tb38913.x

[60] Bao S, Chen L, Thompson RF (1998) Classical eyeblink conditioning in two strains of mice: conditioned responses, sensitization, and spontaneous eyeblinks. Behav Neurosci 112: 714–718. 9676986 10.1037//0735-7044.112.3.714

[61] Gruart A, López-Ramos JC, Muñoz MD, Delgado-García JM (2008) Aged wild-type and APP, PS1, and APP+PS1 mice present similar deficits in associative learning and synaptic plasticity independent of amyloid load. Neurobiol Dis 30: 439–450. 18442916 10.1016/j.nbd.2008.03.001

[62] Woodruff-Pak DS, Logan CG, Thompson RF (1990) Neurobiological substrates of classical conditioning across the life span. Ann N Y Acad Sci 608: 150–173; discussion 74–78. 10.1111/j.1749-6632.1990.tb48896.x 2075952

[63] Woodruff-Pak DS, Papka M, Romano S, Li YT (1996) Eyeblink classical conditioning in Alzheimer’s disease and cerebrovascular dementia. Neurobiol Aging 17: 505–512. 8832623

[64] López-Ramos JC, Jurado-Parras MT, Sanfeliu C, Acuña-Castroviejo D, Delgado-García JM (2012) Learning capabilities and CA1-prefrontal synaptic plasticity in a mice model of accelerated senescence. Neurobiol Aging 33: 627.e13–26. 10.1016/j.neurobiolaging.2011.04.005 21664007

[65] Woodruff-Pak DS, Jaeger ME (1998) Predictors of eyeblink classical conditioning over the adult age span. Psychol Aging 13: 193–205. 9640581 10.1037//0882-7974.13.2.193

[66] Atarashi R, Nishida N, Shigematsu K, Goto S, Kondo T, et al (2003) Deletion of N-terminal residues 23–88 from prion protein (PrP) abrogates the potential to rescue PrP-deficient mice from PrP-like protein/doppel-induced neurodegeneration. J Biol Chem 278: 28944–28949. 12759361 10.1074/jbc.M303655200

[67] Tuzi NL, Gall E, Melton D, Manson JC (2002) Expression of doppel in the CNS of mice does not modulate transmissible spongiform encephalopathy disease. J Gen Virol 83: 705–711. 11842265 10.1099/0022-1317-83-3-705

[68] Chen L, Bao S, Lockard JM, Kim JK, Thompson RF (1996) Impaired classical eyeblink conditioning in cerebellar-lesioned and Purkinje cell degeneration (*pcd*) mutant mice. J Neurosci 16: 2829–2838. 8786457 10.1523/JNEUROSCI.16-08-02829.1996 PMC6578747

[69] Landis SC, Mullen RJ (1978) The development and degeneration of Purkinje cells in *pcd* mutant mice. J Comp Neurol 177: 125–143. 200636 10.1002/cne.901770109

[70] Nolan BC, Freeman JH (2006) Purkinje cell loss by OX7-saporin impairs acquisition and extinction of eyeblink conditioning. Learn Mem 13: 359–365. 16741286 10.1101/lm.168506 PMC1475818

[71] Woodruff-Pak DS, Cronholm JF, Sheffield JB (1990) Purkinje cell number related to rate of classical conditioning. Neuroreport 1: 165–168. 2129873 10.1097/00001756-199010000-00020

[72] Bracha V, Zhao L, Wunderlich DA, Morrissy SJ, Bloedel JR (1997) Patients with cerebellar lesions cannot acquire but are able to retain conditioned eyeblink reflexes. Brain 20: 1401–1413. 10.1093/brain/120.8.1401 9278630

[73] Daum I, Channon S, Canavan AG (1989) Classical conditioning in patients with severe memory problems. J Neurol Neurosurg Psychiatry 52: 47–51. 2496204 10.1136/jnnp.52.1.47 PMC1032655

[74] Dimitrova A, Gerwig M, Brol B, Gizewski ER, Forsting M, et al (2008) Correlation of cerebellar volume with eyeblink conditioning in healthy subjects and in patients with cerebellar cortical degeneration. Brain Res 1198: 73–84. 18262502 10.1016/j.brainres.2008.01.034

[75] Gerwig M, Kolb FP, Timmann D (2007) The involvement of the human cerebellum in eyeblink conditioning. Cerebellum 6: 38–57. 17366265 10.1080/14734220701225904

[76] Topka H, Valls-Sole J, Massaquoi SG, Hallett M (1993) Deficit in classical conditioning in patients with cerebellar degeneration. Brain 116: 961–969. 8353718 10.1093/brain/116.4.961

[77] Chen C, Kano M, Abeliovich A, Chen L, Bao S, et al (1995) Impaired motor coordination correlates with persistent multiple climbing fiber innervation in PKCγ mutant mice. Cell 83: 1233–1242. 8548809 10.1016/0092-8674(95)90148-5

[78] Miyata M, Kishimoto Y, Tanaka M, Hashimoto K, Hirashima N, et al (2011) A role for myosin Va in cerebellar plasticity and motor learning: a possible mechanism underlying neurological disorder in myosin Va disease. J Neurosci 31: 6067–6078. 21508232 10.1523/JNEUROSCI.5651-10.2011 PMC6632970

[79] Rangel A, Madroñal N, Gruart A, Gavín R, Llorens F, et al (2009) Regulation of GABA(A) and glutamate receptor expression, synaptic facilitation and long-term potentiation in the hippocampus of prion mutant mice. PLoS One 4: e7592. 19855845 10.1371/journal.pone.0007592 PMC2763346

[80] Berger TW, Alger B, Thompson RF (1976) Neuronal substrate of classical conditioning in the hippocampus. Science 192: 483–485. 1257783 10.1126/science.1257783

[81] Gruart A, Muñoz MD, Delgado-García JM (2006) Involvement of the CA3-CA1 synapse in the acquisition of associative learning in behaving mice. J Neurosci 26: 1077–1087. 16436593 10.1523/JNEUROSCI.2834-05.2006 PMC6674570

[82] Solomon PR, Solomon SD, Schaaf EV, Perry HE (1983) Altered activity in the hippocampus is more detrimental to classical conditioning than removing the structure. Science 220: 329–331. 6836277 10.1126/science.6836277

[83] Woodruff-Pak DS, Li YT, Hinchliffe RM, Port RL (1997) Hippocampus in delay eyeblink classical conditioning: essential for nefiracetam amelioration of learning in older rabbits. Brain Res 747: 207–218. 9045995 10.1016/s0006-8993(96)01191-2

[84] Kishimoto Y, Kawahara S, Kirino Y, Kadotani H, Nakamura Y, et al (1997) Conditioned eyeblink response is impaired in mutant mice lacking NMDA receptor subunit NR2A. Neuroreport 8: 3717–3721. 9427357 10.1097/00001756-199712010-00012

[85] Kishimoto Y, Kawahara S, Mori H, Mishina M, Kirino Y (2001) Long-trace interval eyeblink conditioning is impaired in mutant mice lacking the NMDA receptor subunit ε1. Eur J of Neurosci 13: 1221–1227. 11285019 10.1046/j.0953-816x.2001.01486.x

[86] Van Der Giessen RS, Koekkoek SK, van Dorp S, De GruijlJR, Cupido A, et al (2008) Role of olivary electrical coupling in cerebellar motor learning. Neuron 58: 599–612. 18498740 10.1016/j.neuron.2008.03.016

[87] Bullock D, Fiala JC, Grossberg S (1994) A neural model of timed response learning in the cerebellum. Neural Netw 7: 1101–1114.

[88] Mauk MD, Donegan NH (1997) A model of Pavlovian eyelid conditioning based on the synaptic organization of the cerebellum. Learn Mem 4: 130–158. 10456059 10.1101/lm.4.1.130

[89] Bao S, Chen L, Qiao X, Knusel B, Thompson RF (1998) Impaired eye-blink conditioning in waggler, a mutant mouse with cerebellar BDNF deficiency. Learn Mem 5: 355–364. 10454360 PMC311271

[90] Qiao X, Chen L, Gao H, Bao S, Hefti F, et al (1998) Cerebellar brain-derived neurotrophic factor-TrkB defect associated with impairment of eyeblink conditioning in Stargazer mutant mice. J Neurosci 18: 6990–6999. 9712667 10.1523/JNEUROSCI.18-17-06990.1998 PMC6792950

[91] Chen L, Bao S, Qiao X, Thompson RF (1999) Impaired cerebellar synapse maturation in waggler, a mutant mouse with a disrupted neuronal calcium channel γ subunit. Proc Natl Acad Sci USA 96: 12132–12137. 10518588 10.1073/pnas.96.21.12132 PMC18424

[92] Hashimoto K, Fukaya M, Qiao X, Sakimura K, Watanabe M, et al (1999) Impairment of AMPA receptor function in cerebellar granule cells of ataxic mutant mouse stargazer. J Neurosci 19: 6027–6036. 10407040 10.1523/JNEUROSCI.19-14-06027.1999 PMC6783074

[93] Herms JW, Korte S, Gall S, Schneider I, Dunker S, et al (2000) Altered intracellular calcium homeostasis in cerebellar granule cells of prion protein-deficient mice. J Neurochem 75: 1487–1492. 10987828 10.1046/j.1471-4159.2000.0751487.x

[94] Herms J, Tings T, Gall S, Madlung A, Giese A, et al (1999) Evidence of presynaptic location and function of the prion protein. J Neurosci 19: 8866–8875. 10516306 10.1523/JNEUROSCI.19-20-08866.1999 PMC6782778

[95] Prestori F, Rossi P, Bearzatto B, Laine J, Necchi D, et al (2008) Altered neuron excitability and synaptic plasticity in the cerebellar granular layer of juvenile prion protein knock-out mice with impaired motor control. J Neurosci 28: 7091–7103. 18614678 10.1523/JNEUROSCI.0409-08.2008 PMC6670502

[96] Porras-García E, Cendelin J, Domínguez-del-Toro E, Vožeh F, Delgado-García JM (2005) Purkinje cell loss affects differentially the execution, acquisition and prepulse inhibition of skeletal and facial motor responses in Lurcher mice. Eur J Neurosci 21: 979–988. 15787704 10.1111/j.1460-9568.2005.03940.x

