# Supplementary figures and images for: Age-Dependent Impairment of Eyeblink Conditioning in Prion Protein-Deficient Mice
Source: PLoS One. 2013 Apr 10;8(4):e60627. doi: 10.1371/journal.pone.0060627 (PMC3622692; doi:10.1371/journal.pone.0060627)

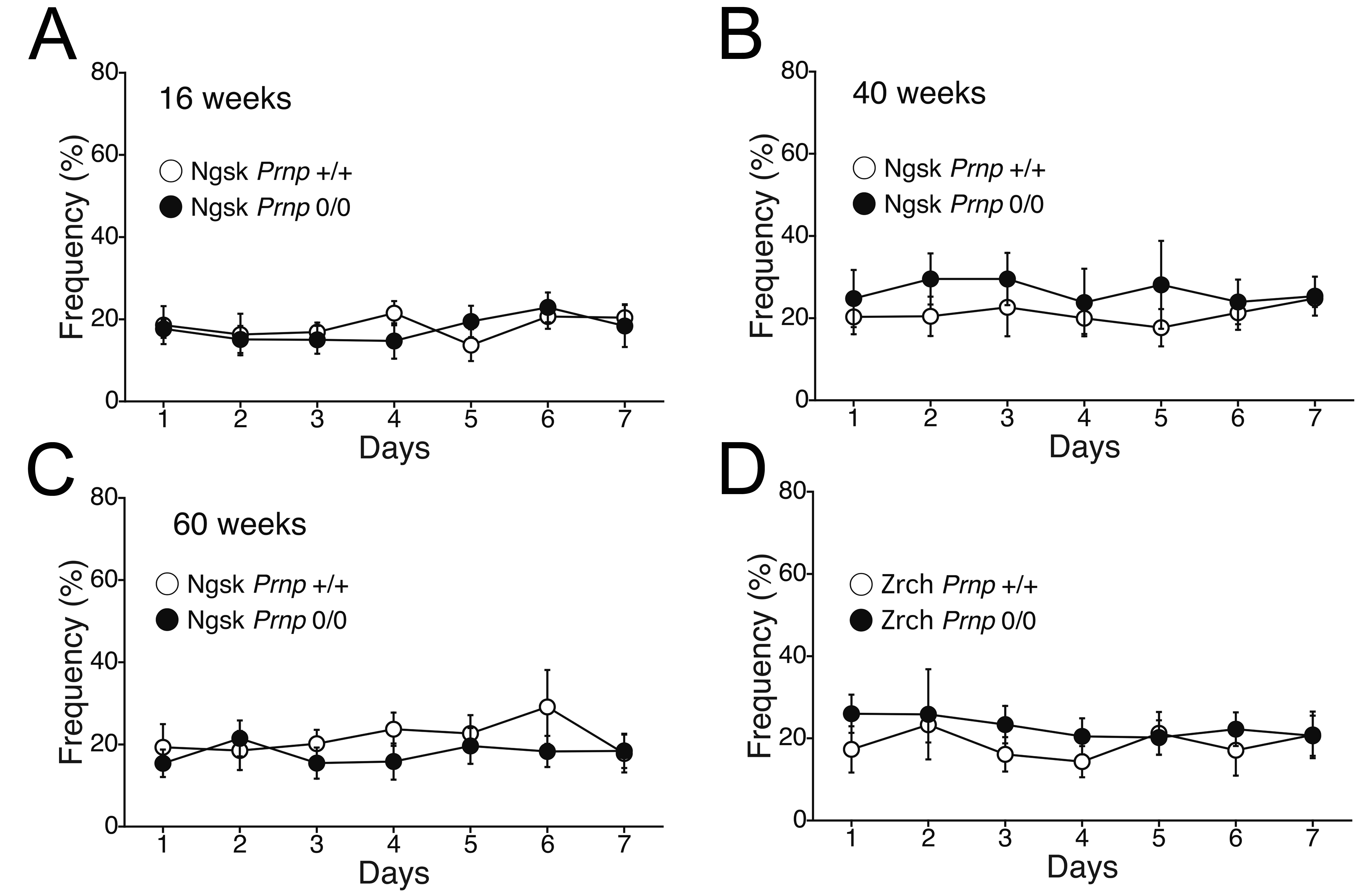

Supplement: Figure S1 — Pseudoconditioning in prion knockout mice. (A) Pseudoconditioning in Ngsk prnp 0/0 (n = 4) and their control (n = 4) mice at 16 weeks old. (B) Pseudoconditioning in Ngsk prnp 0/0 (n = 8) and their control (n = 9) mice at 40 weeks old. (C) Pseudoconditioning in Ngsk prnp 0/0 (n = 8) and their control (n = 8) mice at 60 weeks old. (D) Pseudoconditioning in Zrch prnp 0/0 (n = 10) and their control (n = 9) mice at 60 weeks old. The data points represent the mean ± SEM. (TIF) [file pone.0060627.s001.tif]

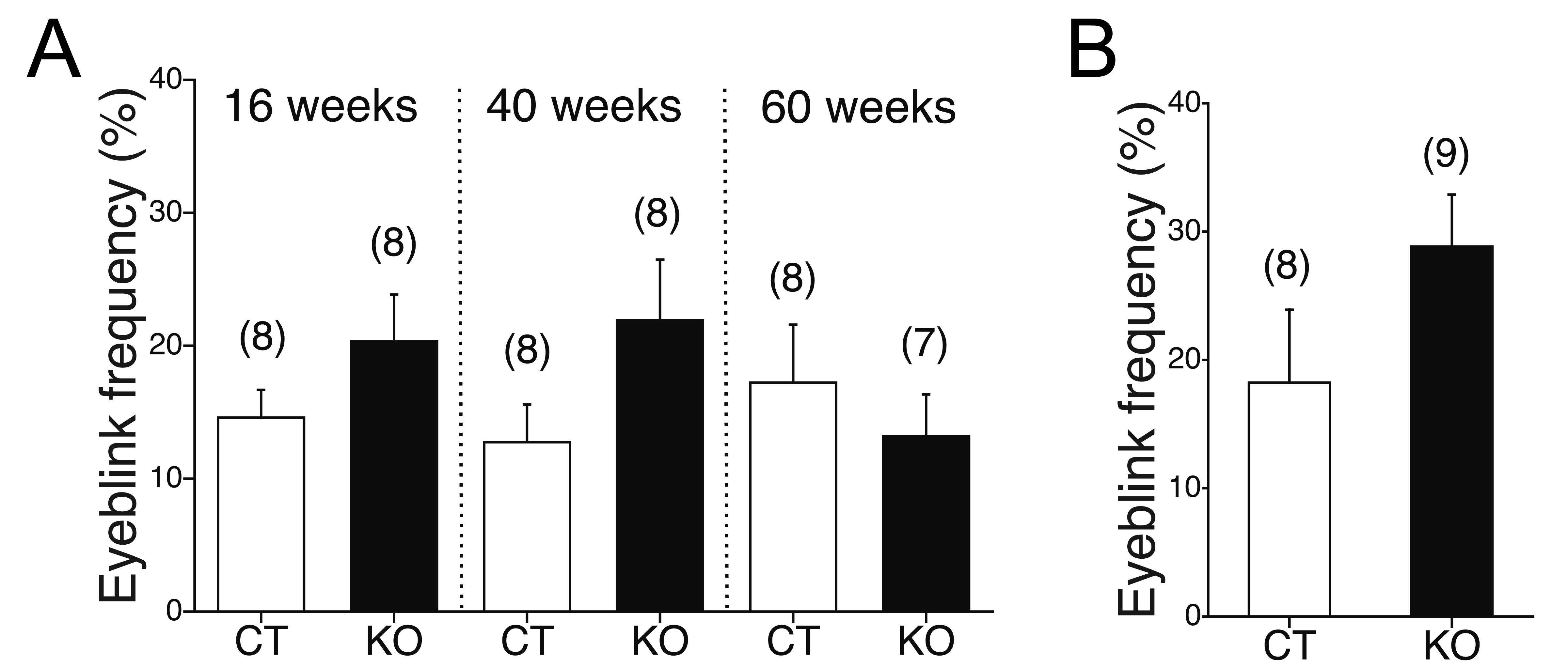

Supplement: Figure S2 — Normal auditory response to tone CS in prion knockout mice. (A) Frequency of eyeblink response during CS in Ngsk prnp 0/0 (KO) and their control (CT) mice at the ages of 16, 40, and 60 weeks. (B) Frequency of eyeblink response during CS in Zrch prnp 0/0 (KO) and their control mice (CT). The data points represent the mean ± SEM. The values in parentheses above the column indicate the number of mice used. (TIF) [file pone.0060627.s002.tif]
