# Supplementary material for: Age-Dependent Impairment of Eyeblink Conditioning in Prion Protein-Deficient Mice
Source: PLoS One. 2013 Apr 10;8(4):e60627. doi: 10.1371/journal.pone.0060627 (PMC3622692; doi:10.1371/journal.pone.0060627)
Supplement: Text S1 — Supplemental Methods and Supplemental Results. (DOCX) [file pone.0060627.s003.docx]

## Title

**Age-dependent impairment of eyeblink conditioning in prion protein-deficient mice**

_____________________________________________________

Supporting Text S1

**Supplemental Methods**

*Animals* Ngsk *Prnp*^0/0^, Zrch *Prnp*^0/0^, and control mice were used in the pseudoconditioning and sensitivity tests. A mixture of 3 wild-type control strains, which included C57BL/6J, 129/SvJ, and FVB/Nj, were used as a control for Zrch *Prnp*^0/0^ mice. Details of these mice are described in the Materials and Methods section. All mice were used only once for each behavioral test, and they were maintained until they reached the ages of 16, 40, or 60 weeks.

*Pseudoconditioning* In the pseudoconditioning paradigm, CS and US were pseudorandomly presented with an interstimulus interval duration ranging from 0 to 20 s. The intertrial interval duration was randomized between 20 and 40 s, with a mean of 30 s. The time windows used for calculation of eyeblink frequency in the test were the same as those used in the CS-only trials of delay eyeblink conditioning [1]. The surgery and stimulus used in the test are basically the same as those used in the delay eyeblink conditioning experiment (See Materials and Methods).

*Tests of the auditory responses* The spontaneous level of eyeblink frequency during the presence of CS was measured by 100 CS-only trials in PrP KO mice. Animals never presented with a US were used in the test. Surgery and CS used in the test are basically the same as those used in the delay eyeblink conditioning experiment (See Materials and Methods). The time windows used for calculation of eyeblink frequency in the test were the same as those used in the CS-only trials of delay eyeblink conditioning (Kishimoto et al., 2002).

*Statistical analysis* Data obtained in the behavioral tests were analyzed using Student’s t-tests or 2-way (age × genotype) analysis of variance (ANOVA), which was followed by a post hoc Scheffe’s test; all analysis was carried out using the SPSS program (IBM Corporation, Armonk, NY), and all of the data are presented as mean ± SEM. Significance was assigned when the *p* value was <0.05.

**Supplemental Results**

*Pseudoconditioning in prion KO mice* To check non-associative responses in prion KO mice, pseudoconditioning with pseudorandomized presentations of CS and US was tested in Ngsk and Zrch *Prnp*^0/0^ mice (Figure S1). First, we tested the pseudoconditioning in Ngsk *Prnp*^0/0^ mice, aged 16 weeks (Figure S1A), but we found no significant interaction effect between sessions and genotypes (*F*_(6,36)_ = 0.54, *p* = 0.774) and no genotype effect (*F*_(1,6)_ = 0.119, *p* = 0.742). Next, we tested pseudoconditioning in Ngsk *Prnp*^0/0^ mice, aged 40 weeks, (Figure S1B). Again, we found no significant interaction effect between sessions and genotypes (*F*_(6,90)_ = 0.292, *p* = 0.934), and no genotype effect (*F*_(1,15)_ = 0.487, *p* = 0.496). Furthermore, with regard to the 60-week-old mice, we found no significant interaction effect between sessions and genotypes (*F*_(6,84)_ = 0.465, *p* = 0.823) and no genotype effect (*F*_(1,14)_ = 3.038, *p* = 0.103) between the two genotypic groups (Figure S1C). Taken together, pseudoconditioning was not altered in Ngsk *Prnp*^0/0^ mice throughout the experiment.

Finally, we evaluated pseudoconditioning in Zrch *Prnp*^0/0^ mice (Figure S1D). We found no significant interaction effect between sessions and genotypes (*F*_(6,102)_ = 0.545, *p* = 0.772), and no genotype effect (*F*_(1,17)_ = 0.798, *p* = 0.384), thus indicating that there was no non-associative factor difference between control and Zrch *Prnp*^0/0^ mice.

*Auditory responses in prion KO mice* With regards to the delay conditioning, the prion KO mice exhibited hyperexcitability to the CS stimuli even at 16 and 40 weeks of age, which is comparable to that exhibited by the control mice (Figures 1, 3, and 5). To confirm whether the CS induced the enhancement of spontaneous eyeblink frequency level in PrP KO mice, we evaluated auditory responses in prion KO mice that were never presented with a US (Figure S2). First, we tested the auditory response of Ngsk *Prnp*^0/0^ mice at the ages of 16, 40, and 60 weeks (Figure S2A). Two-way ANOVA revealed a non-significant main effect of genotype (*F*_(1,41)_ = 1.722; *p* = 0.197) and non-significant effects of age (*F*_(2,46)_ = 0.317; *p* = 0.729), and the interaction effect was not significant (*F*_(2,41)_ = 1.883; *p* = 0.165). Thus, the auditory responses to tone CS were not altered in Ngsk *Prnp*^0/0^ mice at any age examined, even though there was a trend towards higher eyeblink frequencies in the mutant mice at the ages of 16 and 40 weeks.

Next, we evaluated the auditory responses in Zrch *Prnp*^0/0^ mice at the age of 16 weeks (Figure S2B). We again failed to detect a significant difference in the frequency of auditory responses in Zrch *Prnp*^0/0^ mice (*p* = 0.234).

**_______________________________________________________________________**

**Reference for Supporting Text S1**

1. Kishimoto Y, Fujimichi R, Araishi K, Kawahara S, Kano M, et al. (2002) mGluR1 in cerebellar Purkinje cells is required for normal association of temporally contiguous stimuli in classical conditioning. Eur J Neurosci 16: 2416-2424.
